# Relaxometric Studies of Gd-Chelate Conjugated on the Surface of Differently Shaped Gold Nanoparticles

**DOI:** 10.3390/nano10061115

**Published:** 2020-06-05

**Authors:** Parisa Fatehbasharzad, Rachele Stefania, Carla Carrera, Ivan Hawala, Daniela Delli Castelli, Simona Baroni, Miriam Colombo, Davide Prosperi, Silvio Aime

**Affiliations:** 1 Molecular and Preclinical Imaging Centers, Department of Molecular Biotechnology and Health Sciences, University of Torino, 10126 Torino, Italy; p.fatehbasharzad@campus.unimib.it (P.F.); rachele.stefania@unito.it (R.S.); carla.carrera@unito.it (C.C.); ivan.hawala@unito.it (I.H.); simona.baroni@unito.it (S.B.); silvio.aime@unito.it (S.A.); 2 Department of Materials Science, University of Milano-Bicocca, 20126 Milan, Italy; 3 Department of Biotechnology and Bioscience, University of Milano-Bicocca, 20126 Milan, Italy; miriam.colombo@unimib.it (M.C.); davide.prosperi@unimib.it (D.P.)

**Keywords:** gold nanoparticle, concave cube, MRI, contrast agent, gadolinium

## Abstract

Nowadays, magnetic resonance imaging (MRI) is one of the key, noninvasive modalities to detect and stage cancer which benefits from contrast agents (CA) to differentiate healthy from tumor tissue. An innovative class of MRI CAs is represented by Gd-loaded gold nanoparticles. The size, shape and chemical functionalization of Gd-loaded gold nanoparticles appear to affect the observed relaxation enhancement of water protons in their suspensions. The herein reported results shed more light on the determinants of the relaxation enhancement brought by Gd-loaded concave cube gold nanoparticles (CCGNPs). It has been found that, in the case of nanoparticles endowed with concave surfaces, the relaxivity is remarkably higher compared to the corresponding spherical (i.e., convex) gold nanoparticles (SPhGNPs). The main determinant for the observed relaxation enhancement is represented by the occurrence of a large contribution from second sphere water molecules which can be exploited in the design of high-efficiency MRI CA.

## 1. Introduction

The widely-adopted magnetic resonance imaging is an essential modality in clinics for its ability of providing excellent quality images of soft tissues with high spatial and temporal resolution [1]. MRI is highly appreciated in the clinical practice because it allows long term longitudinal studies without the use of any radiotracer (as in single-photon emission computed tomography and positron emission tomography) as well as without the involvement of ionizing radiations (as in X-ray) [2]. Often, in order to enhance the contrast between healthy and pathological tissues, it is possible to make use of contrast agents that markedly increase the relaxation rate of water protons in the region where they distribute [3]. Gadolinium ion is the candidate of choice for yielding a marked enhancement of the relaxation rate of water protons because of its seven unpaired electrons (one in each f orbital) endowed with a remarkably long electron relaxation time [2]. However, Gd(III) ion is toxic as it acts as a potent antagonist of Ca^2+^ ions. Therefore, it has to be administered under the form of a stable chelate to avoid an undesired in vivo release [4]. Nowadays, highly stable paramagnetic complexes of Gd(III) are extensively used in MRI procedures; anytime there is the need for visualizing small tumor lesions or abnormalities in the excretion pathways [5]. One issue with small molecule contrast agents is their low sensitivity, which causes their high doses usage. On the other hand, a number of recent studies have highlighted the use of nanoparticles as contrast agents [6,7,8,9], due to their longer vascular half-life and a higher sensitivity that eventually enables cellular visualization, either via their internalization or via targeting proper epitopes on the outer side of their membranes [10,11]. Gold nanoparticles have attracted attention in the field of bioimaging applications because of their inertness, easy surface modification and excellent stability [12,13]. The chemistry of alkanethiol adsorption onto gold surfaces has been extensively scrutinized [14]. In this regard, several studies have reported the advantages of spherical gold nanoparticles in MRI application [15,16]. However, nanoparticle customization may allow to control their shape, thus introducing the access to investigate properties that are size and shape dependent [17]. Walker and colleagues demonstrate that the chemical properties of organic molecules bound to non-spherical nanoparticles are dependent upon the shape and local curvature of the particles [18]. In this regard, nanostar gold nanoparticles showed that the morphology of the nanoparticles have a marked effect on the relaxivity of surface bound Gd(III) complex [19]. Since the millimolar relaxivity determines the efficiency of the CA in MRI application, finding out the relation between structural features of the nanoparticles and the determinants of contrast agent’s relaxivity is critical. The overall relaxivity of paramagnetic systems consists of three contributions, namely the inner sphere, second sphere and outer sphere relaxivity [3]. One common approach for improving the sensitivity of designed CA is attaching it to proteins or nanoparticles as the relaxivity increases at the imaging fields with the increase of the rotational correlation time (*τ_r_*) [20]. Based on Solomon–Bloembergen–Morgan (SBM) theory, binding the complex to macromolecules, i.e., limiting their freely tumbling, results in an higher relaxivity thanks to the increase of the inner sphere contribution [21,22]. In addition, by loading a large number of Gadolinium complexes per particle, the local concentration of CA is increased, thus yielding solutions that can lead to an improved detection of the targeted tissue. On the other hand, the curvature surface of nanoparticles engages another factor in the molar relaxivity associated to water molecules in the second coordination sphere. This aspect has been recently investigated [19,23]. According to Rotz et al., confinement of water molecules between the nanostar arms, provides a relevant second sphere contribution [19]. However, the polydispersity in size and shape of gold nanostars prevents explicit explanation of the relationship between the gold nanostar morphology and surface-bound complex relaxivity. 

Herein, nano-sized CAs were synthesized by conjugating Gd(III) complexes at the surface of both concave cube and spherical gold nanoparticles which are stabilized with PEG chain. The observed relaxivity appears to be affected through changes in the water diffusion in close proximity to the surface of the hydrophilic nanoparticles. These findings allowed us to identify the main determinants for the observed relaxation enhancement associated with the overall architecture of the gold nanoparticles (Au-NPs).

## 2. Materials and Methods 

### 2.1. Materials

All reagents and solvents were purchased from Sigma-Aldrich (Milan, Italy) unless otherwise noted. Concave cube nanoparticle characterization was performed on 120 keV TEM (Jeol 1010, Tokyo, Japan) and the SEM images for spherical nanoparticles was obtained by 15 keV FE-SEM S9000G–TESCAN (Tescan, Brno-Kohoutovice, Czech Republic). UV/vis/NIR spectra of colloidal solutions were acquired on a UV/VIS spectrophotometer Jenway 6715 (Bibby Scientific Limited, Staffordshire, UK). The mean diameter and surface charge of the NPs were assessed with a Zetasizer NanoZS (Malvern Instruments Ltd., Malvern, UK) operating at a light source wavelength of 633 nm. The *r_1_* (longitudinal proton relaxivity) was calculated by measuring the *T_1_* (longitudinal proton relaxation time) (Bruker MiniSpec60, Bruker Corporation, Billerica, MA, USA). ICP-MS was performed on inductively coupled plasma mass spectrometer (ICP-MS) (Element-2; Thermo-Finnigan, Rodano (MI, Italy). Preparative HPLC (high-performance liquid chromatography) runs were performed with a system equipped with Waters 2767 autosampler and autoinjector, Waters 2525 pumps, Waters 3100 MS Detector and Waters 2998 photodiode array (PDA) detector (Milford, MA, USA). UPLC (ultra-performance liquid chromatography)-analytical characterizations were carried out using a Waters UPLC-H-Class system equipped with Acquity QDa MS detector (Waters, Vimodrone, Italy) and dual-wavelength tunable UV/Vis (TUV) detector. Variable temperature ^17^O-NMR measurements were registered at 14.09T (600 MHz for 1H) on a BRUKER Avance 600 spectrometer.

### 2.2. Synthesis of Gd-DOTAMA-Thiol Complex

**Scheme 1:**
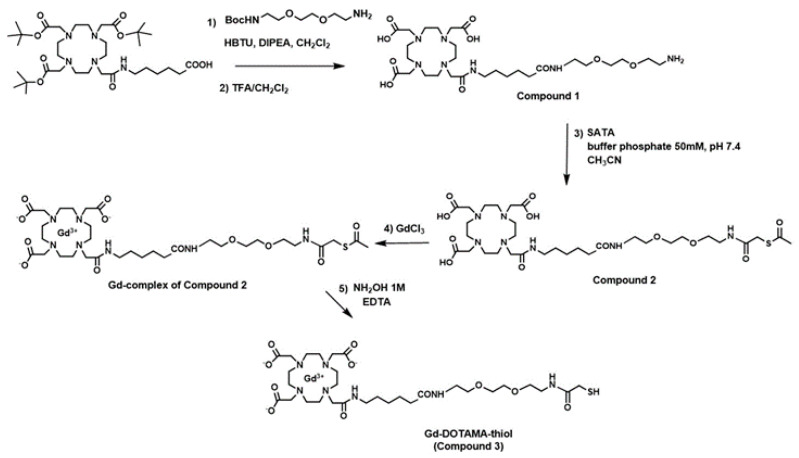
Synthetic pathways to Gd-DOTAMA-thiol (compound **3**).

The overall synthetic pathway is reported in Scheme 1. DOTAMA(*t*BuO)_3_-C_6_-OH was synthesized according to a previously reported procedure [24]. DOTAMA (*t*BuO)_3_-C_6_-OH (0.1 g, 0.14 mmol), Hexafluorophosphate Benzotriazole Tetramethyl Uronium (HBTU, 0.052 g, 0.14 mmol), 1-Hydroxybenzotriazole hydrate (HOBt, 0.020 g, 0.14 mmol) and N,N-Diisopropylethylamine (DIPEA, 0.28 mmol, 50 μL) were dissolved in CH_3_CN. After 15 min mixing, *t*-Boc-N-amido-PEG2-amine was added and allowed to react for 2 h under gentle stirring. The solvent was evaporated to give a yellow solid. The solid was then purified by silica gravimetric direct phase chromatography, with dichloromethane/methanol 9:1. The fractions containing the desired product were collected and combined; the solvent was evaporated to yield a white solid (0.1 g, yield 78%). The cleavage of the *tert-*butyl ester and tert-butyloxycarbonyl groups was done by dissolving the pure product in a solution trifluoroacetic acid (TFA) and Triisopropyl silane (TIS) 95:5 and stirring overnight at room temperature. The deprotected product (compound **1**) was precipitated by adding diethyl ether and kept at 4 °C for 2 h to complete the precipitation. The precipitate was washed with diethyl ether, collected and dried *in vacuo*. The compound **1**. was used in the next step without further purification. SATA reagent (N-succinimidyl S-acetylthioacetate, 0.035 g, 0.15 mmol) was added dropwise to a stirred solution of compound **1** (0.065 g, 0.1 mmol) in 0.05 M phosphate buffer at pH 7.4 and acetonitrile (1:1, 2 mL) and the mixture was stirred at room temperature for 2 h. After evaporation of acetonitrile, the crude product was purified by preparative RP-HPLC using as eluent A, H_2_O containing 0.1% of TFA and as eluent B, CH_3_CN containing 0.1% of TFA with gradient of 5% to 10% of B in 6 min, 10% to 30% of B in 12 min at a flow rate of 20 mL/min and on Atlantis dC18 OBD Prep Column (Waters Corp., Milford, MA, USA), 100Å, 5 µm, 19 mm × 100 mm. UV detection was done at 220 nm. Fractions that contained compound **2** were evaporated and dried in vacuo (0.050 g, yield 60%). The compound **2** was characterized by UPLC-UV-MS using a ACQUITY UPLC BEH C18 column (Waters Corp., Milford, MA, USA, 300Å, 1.7 µm, 2.1 × 100 mm) and 0.05% TFA in water (A) and 0.05% TFA in acetonitrile (B) as solvents, elution initial condition 5% B, linear gradient 5–30% B over 6 min, 30–100% B over 12 min, flow rate 0.4 mL/min and UV detection at 220 nm (*t_R_*, retention time = 3.77 min). The UPLC-UV chromatogram (Figure S1) at 220 nm shows a degree of purity of 96.4% and mass spectrometry of main peak at 3.77 min in the positive ionization mode (ESI +) (Figure S2) indicates a molecular mass of 763.5 g mol^−1^, in agreement with the theoretical molecular weight for C_32_H_57_N_7_O_12_S of 763.4 g mol^−1^. ESI-MS m/z for [M+H]^+^ calculated 764.4, found 764.5; calculated for [M+Na]^+^ 786.4, found 786.5; calculated for [M+2H]^2+^ 383.2, found 382.8.

The Gd (III) complex of ligand 2 was synthesized in water by stoichiometric additions of GdCl_3_ at pH 6.5. The occurrence of residual Gd^3+^ free ion was assessed by UV-vis spectroscopy using the xylenol orange test. The complex containing solution was shown to contain less than 0.3% (mol/mol) of residual free Gd^3+^ ion. The Gd(III) complex was characterized by direct-infusion ESI-MS (+):m/z: calculated for C_32_H_54_GdN_7_O_12_S [M+H]^+^: 919.3, found 919.4; calculated for [M+2H]^2+^: 460.1, found 460.0 (Figure S3). At the final step, 2 mL of 1.0 M hydroxylamine and 50 mM EDTA were added to 1 mL of the complex (0.040 g, 40 mM) in water to remove the acetyl group. The purification step was carried out through gel filtration using a column packed with Sephadex G25 resin (Pharmacia GB Ltd., Hounslow, Middlesex, UK). The recovered Gadolinium complex (Gd-DOTAMA-thiol, compound **3**) containing fraction was lyophilized and stored at −20 °C. The compound **3** was characterized by the UPLC-UV-MS method described above (*t_R_* = 2.8 min). The UPLC-UV chromatogram (Figure S4) shows a *t_R_* = 2.8 min, degree of purity of 95.5%. ESI-MS (+) of main peak at 2.8 min: m/z calculated for C_30_H_52_GdN_7_O_11_S [M+H]^+^ 877.3, found 877.3; calculated for [M+Na]^+^ 899.3, found 899.3; calculated for [M+2H]^2+^ 439.1, found 439.0 (Figure S5).

### 2.3. Synthesis of Concave Cube Nanoparticles

The fabrication of the concave cube gold nanoparticles was carried out by following the protocol reported by Personick et al. [25]. We slightly customized the original procedure to obtain the desired size of nanoparticles. 

The preparation of Au seeds was conducted via rapid injection of 0.60 mL of ice-cold, fresh NaBH_4_ (10 mM), into a strongly stirring solution already containing 0.25 mL of HAuCl_4_ (10 mM) and 10 mL of Cetyltrimethylammonium chloride (CTAC, 100 mM). 

A growth solution was prepared by consecutively adding 0.50 mL of HAuCl_4_ (10 mM), 100 μL of AgNO_3_ (10 mM), 0.20 mL of HCl (1.0 M), then 0.10 mL of AA (100 mM) into 10.00 mL of 0.1 M CTAC plus 100 µL of AgNO_3_ (10 mM). 

**Scheme 2:**
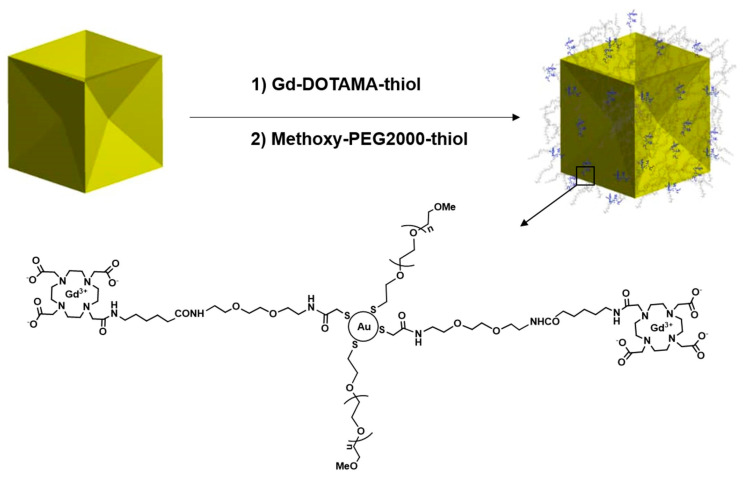
Schematic representation of functionalized CCGNPs (PEG-Gd @CCGNPs).

The seed particles were diluted several times in 0.1 M CTAC to obtain a suspension with 1/80 of the concentration of the mother solution. The particle growth started by adding 0.1 mL of the diluted seeds to the growth solution. The mixture was stirred in a spiral movement immediately after adding the seeds and left to rest on the bench till the completion. The overgrowth of the concave cube gold nanoparticles (CCGNPs) with thin gold films was carried out by using 15 mM gold salt solution. In this system, ascorbic acid reduced the gold in the absence of silver nitrate. The blue-shifted longitudinal plasmon band was also observed for the overgrowth from about 650 nm for the untreated CCGNPs to approximately 620 nm for particles with the thin gold film (8 µM HAuCl4 during the growth). The functionalization of Au NPs was conducted with centrifugation of CCGNPs (10 mL) and re-suspended them in Milli-Q water to remove the extra CTAC. The Gd-DOTAMA-thiol in aqueous solution (3.5 µL, 5.5 mM) was added to the Au colloid under sturdy stirring. After 10 min, in order to avoid the non-specific association of particles to surfaces or dispersed in solution, monofunctional polyethylene glycol (PEG 2kDa) was used to coat nanoparticles’ surface. 1.45 mL of SH-PEG 2000-OMe (10 µM) was added to the stirring solution in one shot. Exchanging the weakly associated CTAC with PEG creates stable gold-thiol dative bonds on the Au surface. Scheme 2 displays the functionalization procedure.

### 2.4. Synthesis of Spherical Nanoparticles

The spherical gold nanoparticle is synthesized by following the procedure reported by Zheng et al. [26] and it was done in three growing steps to obtain the final spherical nanoparticles of 46 nm.

A fresh aqueous NaBH_4_ solution (10 × 10^−3^ M, 0.6 mL) was added to a 10-mL aqueous solution containing HAuCl_4_ (0.25 × 10^−3^ M) and CTAB (100 × 10^−3^ M), in one shot. After adding NaBH_4_, a brown solution was immediately formed. The mixture was shaken for 2 min and then kept at rest at 27 °C for 3 h. In the next step, the synthesized Au cluster (50 µL), in the presence of an aqueous solution of CTAC (200 × 10^−3^ M, 2 mL), and AA (100 × 10^−3^ M, 1.5 mL) was mixed with an aqueous HAuCl_4_ solution (0.5 × 10^−3^ M, 2 mL) and stirred at 27 °C for 15 min. Then the reaction mixture was centrifuged to collect the solid precipitate. The Au nanospheres with a diameter of about 10 nm were dispersed in 1 mL of aqueous CTAC solution (20 × 10^−3^ M) to obtain the product that was used as a seed in the second round of growth. To achieve 46 nm spherical gold nanoparticles, aqueous solutions of CTAC (100 × 10^−3^ M, 2 mL), AA (10 × 10^−3^ M, 130 µL), and the 10 nm seeds (10 µL) were mixed in a 20 mL glass vial, followed by dropwise addition of aqueous HAuCl_4_ solution (0.5 × 10^−3^ M, 2 mL) by a syringe pump at a rate of 2 mL h^−1^. The reaction was allowed to proceed at 27 °C for 10 min after the injection; subsequently centrifugation at 14,500 rpm for 1 h allowed to collect the final product. The functionalization of 46 nm spherical gold nanoparticles was carried out by following the procedure above described for the CCGNPs.

### 2.5. Relaxivity Measurements

A Stelar SpinMaster relaxometer (Stelar S.r.l., Mede (PV) Italy) operating at 0.5 T (21.5 MHz Proton Larmor Frequency) was used to measure the *r_1_* at 25 °C and 37 °C by applying the standard inversion-recovery technique. The obtained values were normalized to 1 mM Gadolinium concentration. The ICP-Ms analysis was applied to detect the Gd concentration.

### 2.6. NMRD Data

The proton 1/*T_1_* NMRD profiles of free and nanoconjugate Gadolinium complex, from 0.01 MHz to 20 MHz Proton Larmor Frequency, were obtained on a fast field-cycling Stelar relaxometer at 25 and 37 °C. The relaxometer operation was controlled by computer with ±1% of an absolute uncertainty in 1/*T_1_*. A Stelar SpinMaster spectrometer with a traditional electromagnet was used to find the data point between 21.5–70 MHz. A Stelar VTC-91 air-flow heater equipped with a copper constantan thermocouple was applied for controlling the temperature with 0.1 °C of uncertainty.

## 3. Results

### 3.1. Characterization of the Gd (III) Complex and Nanoparticles

The synthesis of Gd-DOTAMA-thiol (Scheme 1, compound **3**) was obtained by a first coupling reaction between the DOTAMA(*t*BuO)_3_-C6-OH and the *t*-Boc-N-amido-PEG2-amine and subsequent removal of tert-butyl and N-Boc groups using trifluoroacetic acid and Triisopropylsilane (TIS, 95:5 *v*/*v*). The ligand (compound **1**) was conjugated with SATA before performing complexation step with Gd. Gd(III) complexes of compound **2** were synthesized in water by stoichiometric additions of GdCl_3_ at pH 6.5. The occurrence of residual Gd^3+^ free ion was assessed by UV-vis spectroscopy using the xylenol orange method. Deprotection of –SH group by removing the acetyl group with hydroxylamine was achieved to afford the Gd-complex thiol group completely free (Gd-DOTAMA-thiol) to bind to the surface of Au particles.

The final Gd complex owns a coordination environment, including one amide O donor atom, three carboxylate O donor atoms, and four macrocyclic N donor atoms. The highly thermodynamically stable and kinetically inert Gd (III) complex [16] was characterized by relaxometric analysis. The obtained *r_1_* values measured at 20 MHz, 5.87 mM^−1^ s^−1^ at 298 K is fully consistent with the relaxivities of analogous Gd-DOTAMA complexes reported in the literature [24].

Two types of Au-NPs have been considered for binding the Gd(III) complexes on their surface, namely, (i) concave cube Au-NPs and (ii) spherical (i.e., convex) Au-NPs. The confirmation of nanoparticles morphology and size were conducted by using UV-Visible spectroscopy and dynamic light scattering (DLS). The hydrodynamic diameter of nanoparticles was evaluated both after and before functionalization. The transmission electron microscopy (TEM) was used to reveal the exact shape of CCGNPs.

**Figure 1:**
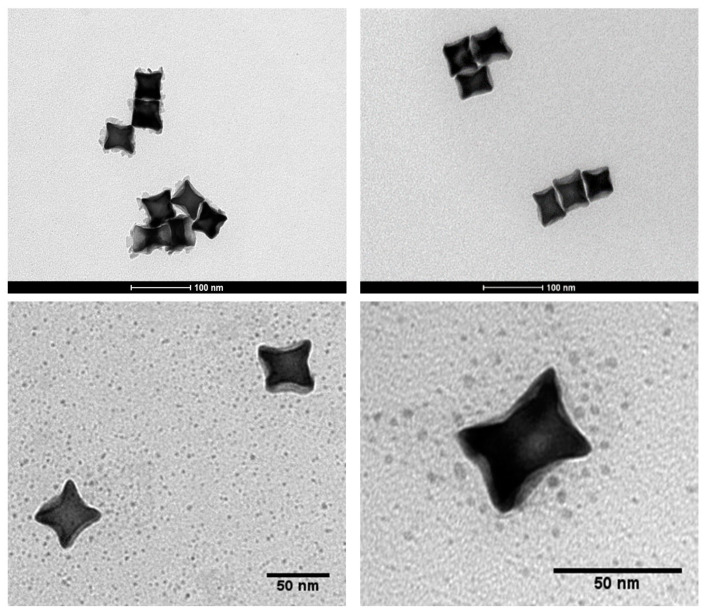
Transmission electron microscopy (TEM) images of concave cube gold nanoparticles.

**Figure 2:**
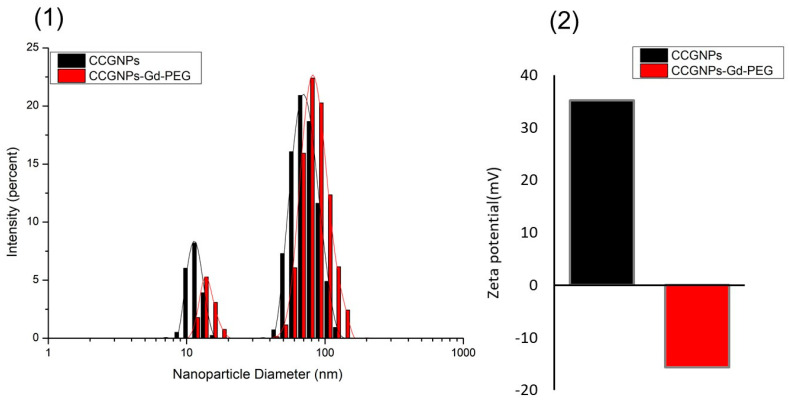
(**1**) Particles size distribution and (**2**) zeta potential of concave cube gold nanoparticles (CCGNPs) before and after PEGylation.

Based on transmission electron micrographs (Figure 1), a mean diameter of CCGNPs was ca. 45 nm from corner to corner. Au concave nanocubes were demonstrated to be highly monodisperse in particle shape, as well as size. In DLS analysis, CTAC-capped CCGNPs displayed two relatively narrow size distribution peaks, one for larger particle sizes around 68–78 nm, and the other one for the smaller sizes, around 13 nm (Figure 2(1)). Based on recent studies, the small particle size distribution is not attributed to real particle size but may represent the rotational diffusion of anisotropic nanoparticles. These artifacts of DLS method arise from the rotational diffusion contribution to the correlation function of the scattered intensities of nonspherical CCGNPs particles; which is accordingly equal to the translation diffusion coefficient for spherical nanoparticles having mean diameters of 13 nm [27,28]. Upon functionalization with Gd-DOTAMA-thiol and with PEG2000, the average hydrodynamic diameter of concave nanocubes was slightly bigger than bare ones and Z-potential changed from +35.2 to −15.6 (Figure 2(2)).

**Figure 3:**
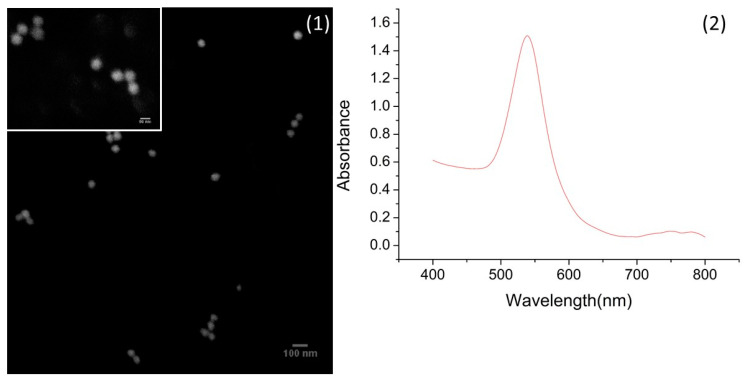
(**1**) Scanning electron microscope (SEM) images of 46-nm spherical nanoparticles and (**2**) UV-vis spectra.

SEM images of spherical gold nanoparticles (SPhGNPs) displayed the homogeneous shape and size of these nanoparticles (Figure 3(1)). Figure 3(2) showed a UV-vis extinction spectra of 46 nm Au nanospheres around 540 nm which proved the complete formation of SPhGNPs [26]. The results from DLS analysis of Au nanospheres are reported in Figure S6. The size distribution of Au nanospheres, around 68–78 nm, appear well consistent with related data reported in the literature [26]. After functionalization of SPhGNPS, the size distribution of the particles was shifted to higher values (Figure S6-1) and the zeta potential changed from +17.5 to −17.4 upon PEGylation.

### 3.2. Determination of Water Exchange Rate

**Figure 4:**
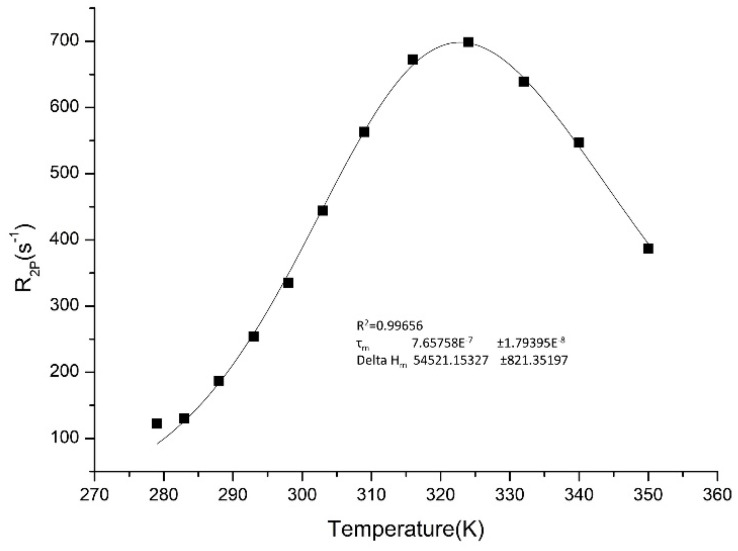
^17^O nuclear magnetic resonance (NMR) measurement of the Gd-DOTAMA-thiol (compound **3**).

One of the important factors to know about the characteristics of potential CA is the water exchange rate *k_ex_* = 1/*τ_m_*. The residence lifetime of water protons *τ_m_*, affects the proton relaxivity by two ways. While it contributes to the chemical exchange of water molecules between the inner sphere and the bulk, it also may be relevant in the overall correlation time, *τ_c_*, that control the electron and nuclear spin dipolar interaction. The accurate assessment of the exchange lifetime of the coordinated water molecule can be pursued by measuring the water ^17^O transverse relaxation rate at variable temperature. Enhancement of relaxation rate for the paramagnetic transverse ^17^O, 1/*T_2r_*, is associated with the water residence time, *τ_m_*, as well as the bound water transverse relaxation rate, 1/*T_2m_*. For the Gd-complex synthesized in this work, the plot of ^17^O-R_2_p (paramagnetic contribution to the transversal relaxation rate of the water oxygen) versus the temperature is reported in Figure 4. By fitting the graph to the established theory [29,30], the value of *τ_m_* was found to be 766 ns at 298 K. This value was fixed in the following data analysis related to NPs, taking into account a possible *τ_m_* modification (less than 3%) by passing from the free complex to the particle.

### 3.3. NMRD Analysis

**Figure 5:**
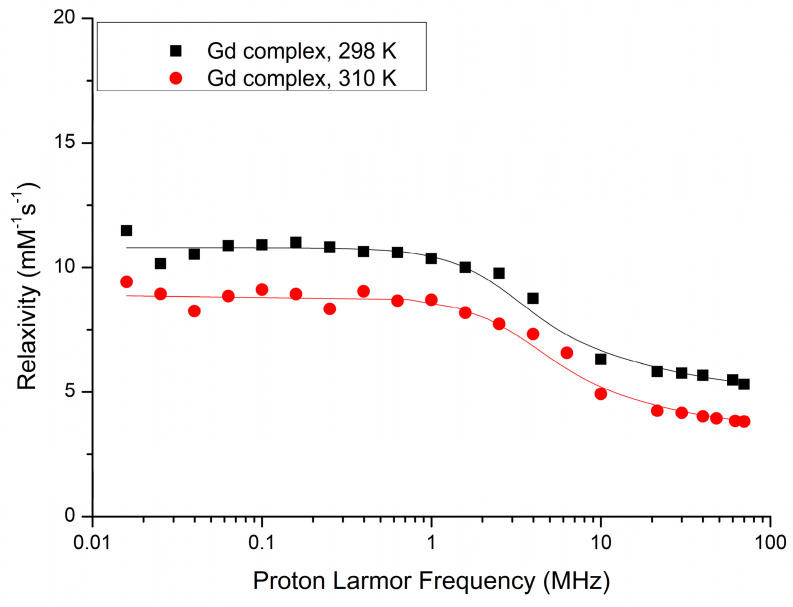
Nuclear magnetic relaxation dispersion (NMRD) profile of Gd-DOTAMA-thiol (compound **3**) at 298 K and 310 K.

To extract the main determinants of the observed proton relaxation enhancement, the nuclear magnetic relaxation dispersion (NMRD) profile for free compound **3** was acquired and fitted to the relevant equations obtained by the established Solomon–Bloembergen–Morgan (SBM) theory (see the Supplementary Materials for the equations) [21,22]. As far as the outer sphere contribution is concerned, we followed the model early described by Hwang and Freed [31]. Some parameters were fixed in the fitting procedure, namely the distance between Gd and the protons of water molecules in the inner and second coordination spheres, number of coordinated water molecules (*n*) and *τ_m_* obtained from ^17^O measurement. An excellent fitting between the experimental and calculated values for the profiles at 298 K and 310 K was achieved (Figure 5, Table 1).

*Δ*: trace of the zero field splitting (ZFS) tensor; *τ_v_*: correlation time for electron relaxation; *τ_r_*: reorientational correlation time, *τ_m_*: water exchange correlation time; n: number of water molecules in the inner coordination sphere; *r*: distance between inner sphere water protons and the gadolinium ion; *a*: distance of closest approach of the solvent protons and the paramagnetic centre; *D*: diffusion coefficient.

**Figure 6:**
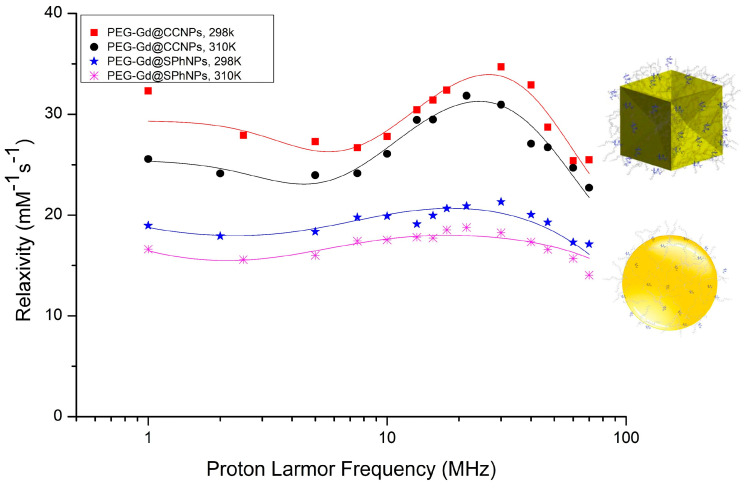
NMRD profile of concave cube and spherical gold nanoparticles at 298 K and 310 K.

These values were compared with the parameters obtained from the NMRD profiles for the Gd-containing nanoparticles. Figure 6 reports the fitted NMRD profiles of the Gd-loaded Au-NPs at 298 K and 310 K, respectively. The relaxation data were normalized to the concentration of Gd(III) complex to analyze the resulting relaxivity profiles as done above for the parent complexes. As the SBM theory does not work properly at low magnetic fields for slow rotating objects, only frequencies above 1 MHz were fitted [16]. The parameters for electronic relaxation were used as empirical fitting parameters and have no real physical meaning. First, we tried to fit the NMRD graphs without any second sphere contribution, but the NMRD profile related to concave cube nanoparticles could not be fitted. Once again, the analysis was carried out considering the presence of a second sphere contribution, leaving the related relevant parameters free to change. Accordingly, the data fitting was performed successfully with remarkable second-sphere water molecules contribution at a distance of 3.5 Å for Gd(III)-proton [19] in the NMRD profile of PEG-Gd@CCGNPs. The NMRD profile showed a maximum relaxivity peak of 34 mM^−1^ s^−1^ at 298 K, centered at approximately 26 MHz, i.e., nearly six-fold the value measured for the free complex. In most Gd(III) complexes, the contribution of second-sphere water molecules is usually incorporated in *r_1_* and it is considered to account for less than 10% [19]. Thus, the herein observed large contribution appears rather a peculiarity that can be associated with the bonding at the surface of these Au-NPs. The analysis suggests a unique hydrophilic environment provided by the PEG-Gd@CCGNPs shape that is evidenced by the occurrence of a large second-sphere contribution. In principle the relaxivity of concave cube nanoparticles might be even higher, and we surmise that the flexibility of the linker used to bind the chelate to the nanoparticle could have hampered to some extent the expected relaxation enhancement. In order to get more insight into this behavior, the observed NMRD profile was analyzed by means of the application of the Lipari–Szabo approach [32] that accounts for the presence of a localized motion superimposed to the overall reorientational motion of the system. However, the obtained results do not add any support to this view as it was found that only one rotational correlation time appeared relevant (the global one) to describe the molecular reorientational motion of the Gd-water vector, as anticipated by applying the SBM treatment.

The suggestion that the high relaxivity observed for the concave cube nanoparticles has to be related to the peculiar shape of these particles was further supported by comparing their NMRD profiles with those ones obtained from the analogous systems based on the spherical particles, i.e., the spherical NPs of 45 nm containing the same Gd(III) complex and PEG. The determinants of the NMRD profile of these two differently shaped gold nanoparticles are compared in Table 2. Both systems display analogous behavior in relation to the temperature, to suggest an overall dominance of the molecular reorientational time in the determination of the observed relaxivities. However, the spherical Au-NPs display a remarkably lower relaxivity peak *r_1_* (20.6 mM^–1^ s^–1^ at 298 K). From the fitted data reported in Table 2, the difference in *r_1_* between the two Gd-containing Au-NPs can be ascribed to the occurrence of a large number of second coordination sphere water molecules in the PEG-Gd@CCGNPs system.

## 4. Discussion

The relaxivity of paramagnetic Gd(III) chelates is the result of a complex interplay of many parameters governing the dipolar interactions between water and the paramagnetic Gd centers. A well-established approach considers three contributions to the observed relaxivity: (1) inner-sphere relaxation, i.e., the metal-coordinated water transmits the relaxation effect to the bulk water being exchanged with the solvent water molecules; (2) second-sphere relaxation, due to water molecules in the second coordination sphere hydrogen-bonded or due to exchangeable hydrogen atoms (such as the N-H in the carboxyamide group) on the ligand’s structure; (3) outer-sphere relaxation, due to the diffusion of water molecules in the proximity of the paramagnetic compound [3]. Based on the SBM theory, the inner sphere relaxivity depends on several parameters such as the number of inner-sphere water molecules, the residence time of the inner-sphere water molecule(s), and the tumbling rate of the paramagnetic complex in solution [33].

Ongoing from the free complex to the Gd-anchored systems, *r_1_* is first of all affected by the change in the rotational correlation time, *τ_r_.* The slowly tumbling nanoparticles induce a marked elongation of the molecular reorientational time (see Table 1 and Table 2) for the Gd (III) complexes bound to the surface of either concave cube or spherical NPs. This results in enhanced *r_1_* relaxivity. However, CCGNPs display a larger relaxivity with respect to SPhGNPs even though their *τ_r_* are comparable.

**Figure 7:**
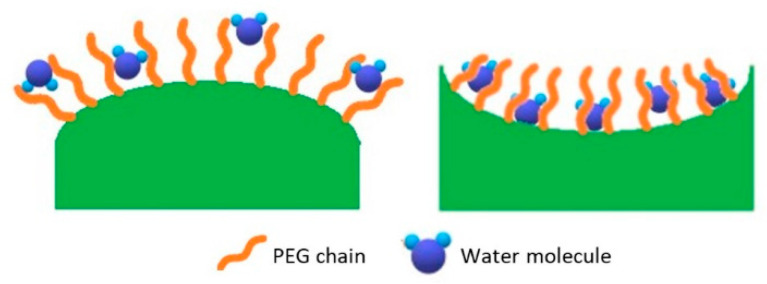
Water distribution on nanoparticles with varying curvatures.

Our work was aimed at getting more insight into the relationship between the observed relaxivity and the shape of the Au-NPS. In nanometer systems, almost all properties are shape- and size-dependent. The morphology of nanoparticles has been chemically controlled by the applied synthetic strategies to provide desired optical, electrical, catalytic and magnetic properties. Specifically, Au NP surface curvature appears to have an influence on the physicochemical properties of conjugated moieties (Figure 7). The effect of shape on the enhancement of the transverse relaxivity (*r_2_*) was previously observed for superparamagnetic nanoparticles such as iron oxide [34]. Similarly, the anisotropic shape of Au-NPs was suggested to play a role in enhancing the longitudinal relaxivity of NP-conjugated Gd(III) MRI CAs. In this context, rod-like and star-like shapes were investigated so far, confirming the assumption that nanoparticle morphology critically affects the *r_1_* of surface-bound ligands [19,35]. In particular, Rotz et al. reported that in gold nanostars the observed high relaxivity was determined by its peculiar shape [19]. However, as-synthesized Au nanostars are generally polydisperse in size and shape, which limits detailed assessment of the relationship between structural features of nanoparticles and their effect on CAs power. Thus, although Gd(III)-conjugated NP have shown established size-dependent *r_1_* enhancement based on *τ_r_* effects [36,37], only a few studies have been conducted to examine the impact of particle shape and surface curvature on *r_1_*. According to Culver and colleagues, the shape of nanoparticles is primarily responsible for influencing *r_1_*, rather than the size [23]. The presently investigated concave cubes are nanocubes with six depressed square faces which make a square pyramid shape, yielding a 24-facet nanostructure. Herein, it has been found that the change in shape between the two considered NPs mostly affects the second sphere contribution. Indeed, the results of NMRD analysis suggested that the surface curvature and shape of nanoparticles can affect the positioning of conjugated PEGs on the surface of particles and might significantly “sequester” water molecules that, in turn, provide a large array of protons in the close proximity of the Gd(III) complexes. The PEG-Gd@SPhGNPs and PEG-Gd@CCGNPs are different only in shapes. In the context of our relaxometric investigations, Gd-containing moieties bound to spherical NPs do not display second-sphere enhancement. The positive curvature (convex surface), makes larger average distance between the head groups of the molecules forming the SAM (self- assembled monolayer); while the negative curvature (concave surface) forms the nanocage- like area well suited to entrap more water molecules (Figure 7). Our interpretation is that this is the reason why water molecules contribute to relaxivity only in the case of CCGNPs second sphere. In fact, the water molecules entrapped between PEG chains are closer to the Gd complex. 

## 5. Conclusions

The variation of particle shape can influence nanoconjugate CA surface dynamics and improve the *r_1_* value through enhancing second-sphere contributions to the CA performance. The present study showed that irregularly shaped nanoparticles, in addition to elongating the rotational correlation time of gadolinium complexes conjugated on their surfaces, are able to affect the second sphere contribution according to the surface curvature. This effect provides a relaxivity 13 times higher than that of currently used clinical contrast agents [38]. A high relaxivity has been observed for a PEG-Gd(III) gold nanoconjugate platform associated with concave cube gold nanoparticles, which makes them promising candidates for MRI contrast agents.

## Figures and Tables

**Table 1:** NMRD best fit parameters derived from NMRD profile of Gd-DOTAMA-thiol (compound **3**) at 298 K and 310 K (Figure 5).

	*S* *	*Δ^2^* (s^−2^)	*τ_v_* (ps)	*τ_r_* (ps)	*τ_m_* (ns) *	*a* (Å) *	*D* (cm^2^ s^−1^) *
298 K	3.5	10^19^ (±4 × 10^18^)	37 (±13)	126 (±7)	770	3.8	2.24 × 10^−5^
310 K	3.5	10^19^ (±7 × 10^18^)	29 (±18)	81 (±6)	735	3.8	3.1 × 10^−5^

Fixed parameters: *; *r* = 3.1 Å; *a* = 3.8 Å; *n* = 1 [24].

**Table 2:** NMRD best fit parameters for PEG-Gd@CCGNPs and PEG-Gd@SPhGNPs at 298 K and 310 K.

	CCGNPs 298 K	CCGNPs 310 K	SPhGNPs 298 K	SPhGNPs 310 K
*S* *	3.5	3.5	3.5	3.5
*Δ*^2^ (s^−2^)	2.3 × 10^19^ (±6.2 × 10^18^)	1.8 × 10^19^ (±3.5 × 10^18^)	3.2 × 10^18^ (±5.8 × 10^17^)	2.8 × 10^18^ (±5.9 × 10^17^)
*τ_v_* (ps)	42.1 (±6.5)	47.2 (±3.8)	23.2 (±4.7)	30.3 (±9.4)
*τ_r_* (ns)	15.4 (±6.6)	11 (±3.1)	3.2 (±0.5)	1.6 (±0.1)
*τ_m_* (ns) *	750	730	750	735
*r* (Å) *	3.1	3.1	3.1	3.1
*n* *	1	1	1	1
*a* (Å) *	3.8	3.8	3.8	3.8
*D* (cm^2^ s^−1^) *	2.24 × 10^−5^	3.1 × 10^−5^	2.24 × 10^−5^	3.1 × 10^−5^
*n*_1_	7.9 (±1.6)	5.6 (±1.1)	-	-
*r*^ss^ (Å) *	3.5	3.5	-	-
*τ*^ss^ (ps)	106.9 (±22)	118.6 (±24.6)	-	-

* Fixed parameters. *Δ*: trace of the Zero Field Splitting (ZFS) tensor; *τ_v_*: correlation time for electron relaxation; *τ_r_*: reorientational correlation time, *τ_m_*: water exchange correlation time; *n*: number of water molecules in the inner coordination sphere; *r*: the distance between inner sphere water protons and the gadolinium ion; *a*: distance of closest approach of the solvent protons and the paramagnetic center; *D*: diffusion coefficient; *n*_1_: number of second sphere water molecules; *r*^ss^: the distance between second sphere water protons and the gadolinium ion; *τ*^ss^: reorientational correlation time related to the second sphere water molecules.

## Supplementary Materials

The following are available online at https://www.mdpi.com/2079-4991/10/6/1115/s1, Figure S1: Chromatogram UPLC-UV at 220 nm of compound **2**; Figure S2: ESI (+) mass spectrum of peak at 3.77 min (compound **2**); Figure S3: ESI (+) mass spectrum by direct-infusion of Gd-complex of compound **2;** Figure S4: Chromatogram UPLC-UV of Gd-DOTAMA-thiol (compound **3**); Figure S5: ESI (+) mass spectrum of main peak at 2.8 min in ESI (+), Gd-DOTAMA-thiol (compound **3**); Figure S6: (1) Particles size distribution and (2) zeta potential of spherical gold nanoparticles (SPhGNPs) before and after PEGylation. Summary of paramagnetic relaxation theory for NMRD profile fitting.
