# Supplementary material for: Relaxometric Studies of Gd-Chelate Conjugated on the Surface of Differently Shaped Gold Nanoparticles
Source: Nanomaterials (Basel). 2020 Jun 5;10(6):1115. doi: 10.3390/nano10061115 (PMC7353348; doi:10.3390/nano10061115)
Supplement: Supplementary file 1 [file nanomaterials-10-01115-s001.pdf]

Supplementary Materials

Relaxometric Studies of Gd-Chelate Conjugated on the Surface of Differently Shaped Gold Nanoparticles

Parisa Fatehbasharzad [1,2], Rachele Stefania [1], Carla Carrera [1], Ivan Hawala [1], Daniela Delli Castelli [1,*], Simona Baroni [1], Miriam Colombo [3], Davide Prosperi [3] and Silvio Aime [1]

1 Molecular and Preclinical Imaging Centers, Department of Molecular Biotechnology and Health Sciences, University of Torino, 10126 Torino, Italy; p.fatehbasharzad@campus.unimib.it (P.F.); rachele.stefania@unito.it (R.S.); carla.carrera@unito.it (C.C.); ivan.hawala@unito.it (I.H.); simona.baroni@unito.it (S.B.); silvio.aime@unito.it (S.A.)

2 Department of Materials Science, University of Milano-Bicocca, 20126 Milan, Italy

3 Department of Biotechnology and Bioscience, University of Milano-Bicocca, 20126 Milan, Italy; miriam.colombo@unimib.it (M.C.); davide.prosperi@unimib.it (D.P.)

* Correspondence: daniela.dellicastelli@unito.it

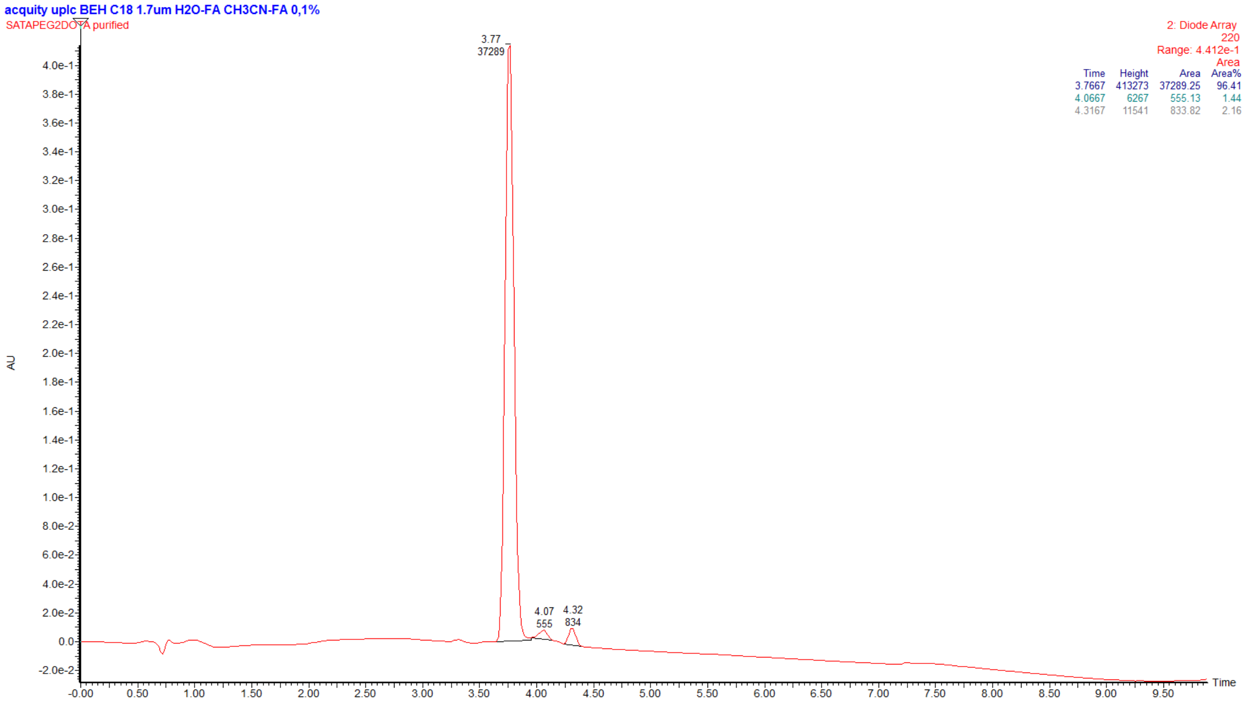

Figure S1. Chromatogram UPLC–UV at 220 nm of compound 2.

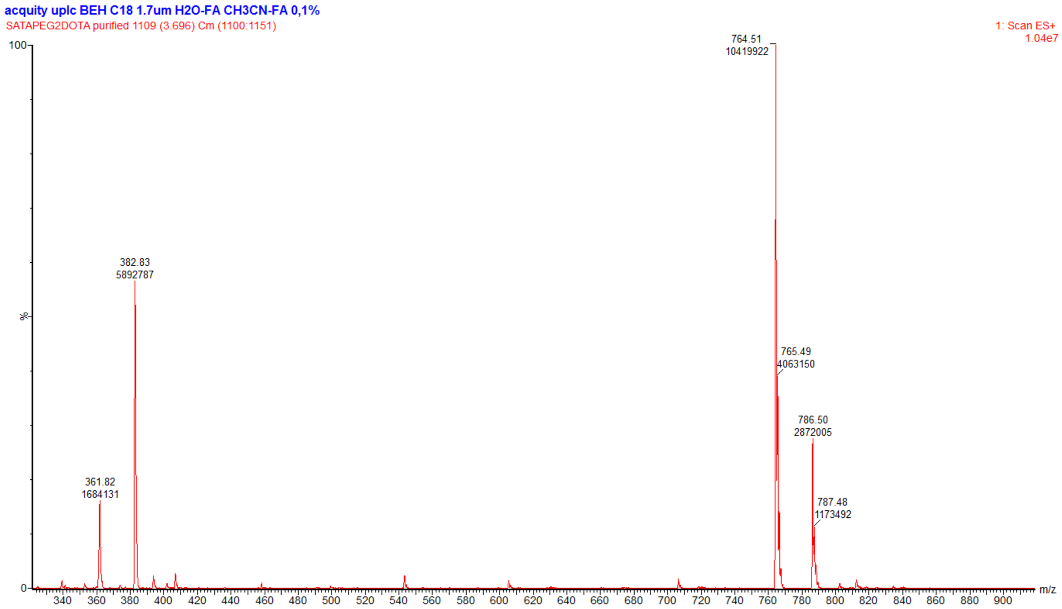

Figure S1. ESI (+) mass spectrum of peak at 3.77 min (compound 2).

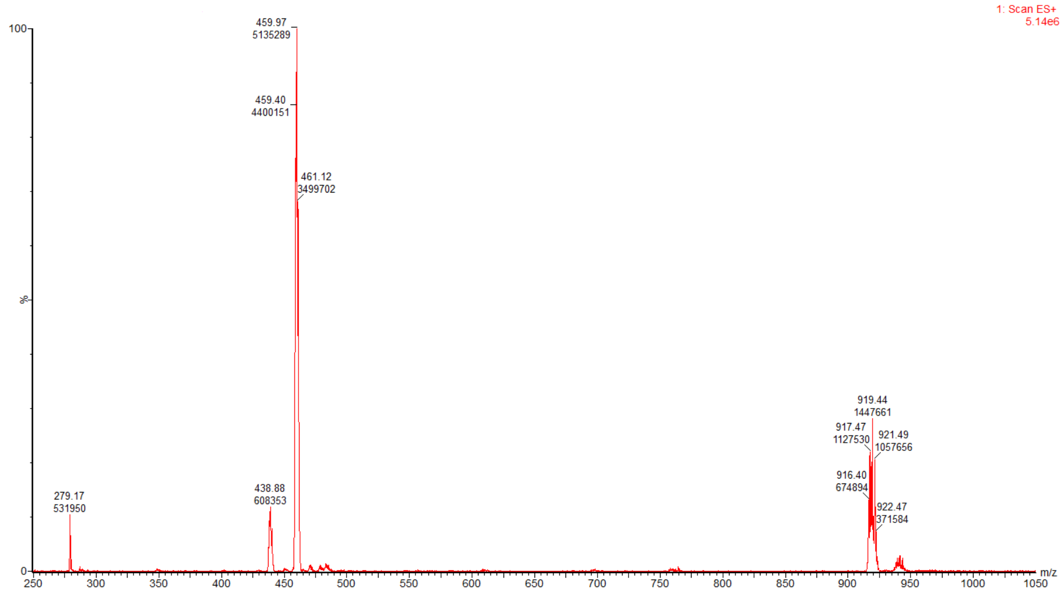

Figure S2. ESI (+) mass spectrum by direct-infusion of Gd-complex of compound 2.

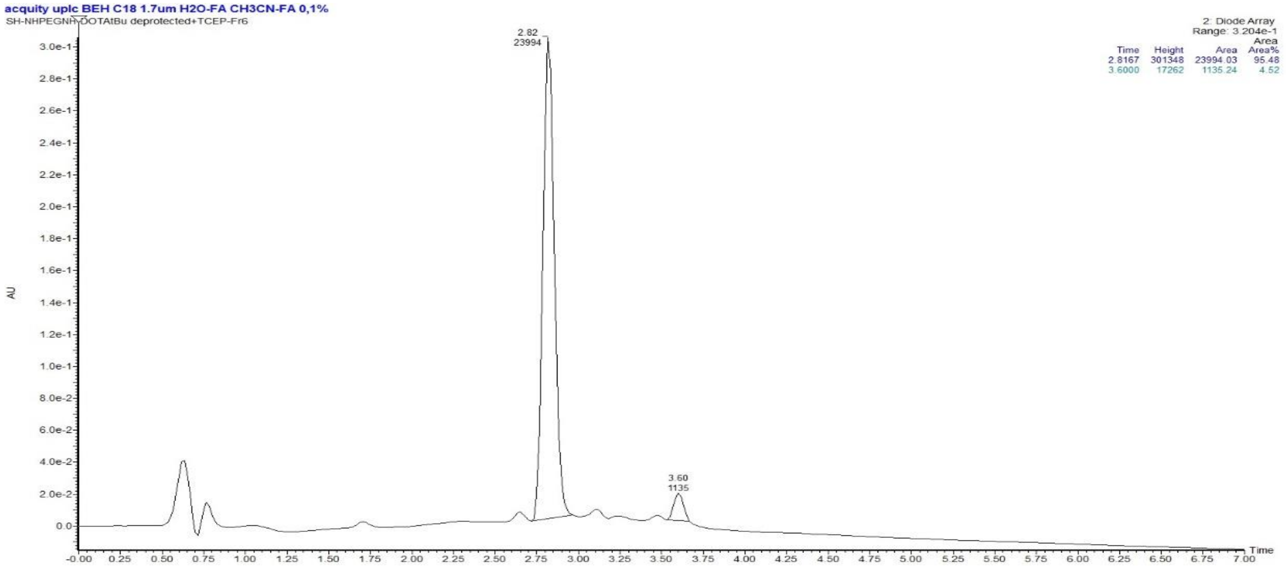

Figure S4. Chromatogram UPLC-UV of Gd-DOTAMA-thiol (compound 3).

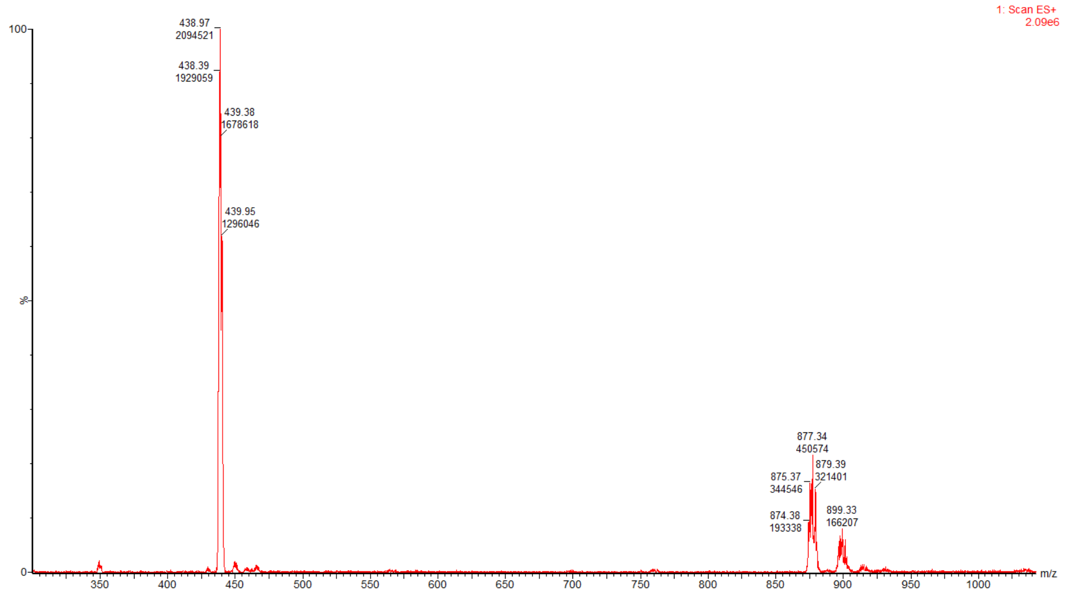

Figure S5. ESI (+) mass spectrum of main peak at 2.8 min in ESI (+), Gd-DOTAMA-thiol (compound 3).

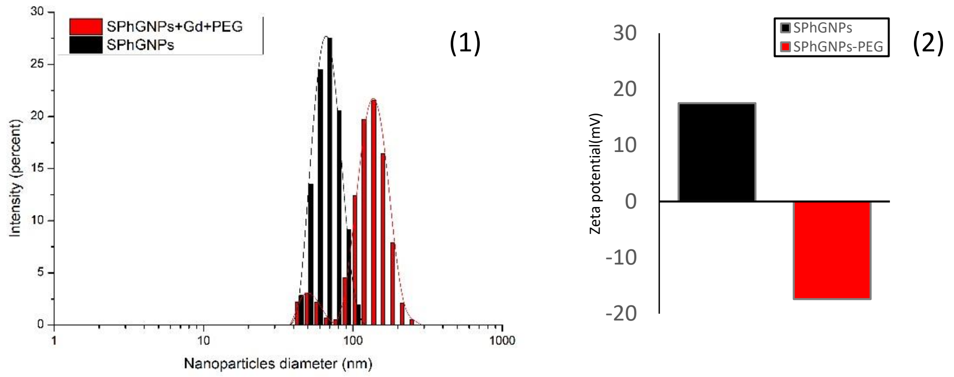

Figure S6. (1) Particle size distribution and (2) zeta potential of SPhGNPs before and after PEGylation.

Summary of Paramagnetic Relaxation Theory for NMRD Profile Fitting

In the presence of Gd-complexes, the observed water proton relaxation rate, $(1/T_1)_{obs}$, is the sum of two contributions:

$$(1/T_1)_{obs} = (1/T_1)_d + (1/T_1)_p \quad (1)$$

where $(1/T_1)_d$ is the (diamagnetic) water relaxation rate in the absence of the paramagnetic species and $(1/T_1)_p$ represents the additional paramagnetic contribution [1]. In the absence of solute–solute interactions, the water relaxation rates are linearly dependent on the concentration of paramagnetic species, [C]. Relaxivity, r_1, is defined as the slope of this dependence in units of $mM^{-1}\ s^{-1}$:

$$(1/T_1)_{obs} = (1/T_1)_d + r_1[C] \quad (2)$$

The field dependence of T_1 by B_0 is represented in the nuclear magnetic resonance dispersion (NMRD) profiles that report about the changes in ($1/T_1$) on function the applied magnetic field strength. The relaxivity enhancement of water protons in the aqueous solutions of paramagnetic complexes arises from time fluctuation of the dipolar coupling between the electron magnetic moment of the metal ion and the nuclear magnetic moment of the solvent nuclei. The dipolar interaction involves both the water molecules that belong to the inner coordination sphere of the metal ion (*inner sphere* contribution, *IS*) and those water molecules that are not directly bound to the paramagnetic center but are either diffusing next to the complex (*outer sphere* contribution, *OS*) or localized in a well-defined position with respect to the metal ion via hydrogen bonding interactions with polar groups of the ligand (*second sphere* contribution, *SS*). Therefore, the total relaxivity is expressed as the sum of the three contributions:

$$r_1 = r_1^{IS} + r_1^{OS} + r_1^{SS} \quad (3)$$

The **inner sphere** longitudinal relaxivity is given by the following expression:

$$r_1^{IS} = \frac{n[C]}{55.6(T_{1M} + \tau_M)} \quad (4)$$

where q is the hydration number, *[C]* is the molar concentration of the paramagnetic species, T_{1M} is the longitudinal relaxation time of the inner-sphere water protons and τ_M is their residence lifetime. The **Solomon–**

Bloembergen theory provides the magnetic field dependence of T_{1M} which, in the case of Gd complexes, is mainly due to the dipolar contribution, defined as follow:

$$\frac{1}{T_{1M}} = \frac{2}{15}\frac{\gamma_H^2 g_e^2 \mu_B^2 S(S+1)}{r_H^6}[3J(\omega_I;\tau_{c1}) + 7J(\omega_S;\tau_{c2})] \quad i = 1,2 \tag{5}$$

where S is the electron spin quantum number, γ_H is the proton nuclear magnetogyric ratio, μ_B is the Bohr magneton, g_e is the Landé factor for the free electron, r_H is the distance between the metal ion and the inner-sphere water protons; ω_I and ω_S are the proton and electron Larmor frequencies ($\omega_S = 658.21 \cdot \omega_I$), respectively, and $J(\omega_i,\tau_i)$ are the spectral density functions, where τ_{ci} (i = 1,2) are the correlation times related to the modulation of the dipolar electron–proton coupling:

$$\tau_{ci}^{-1} = \tau_M^{-1} + \tau_R^{-1} + T_{iE}^{-1} \quad i = 1,2 \tag{6}$$

T_{iE} is the electron spin relaxation time, τ_M is the water residence time as mentioned above, and τ_R is the rotational tumbling time of the entire metal-water unit.

Analogously to the nuclear relaxation time, the electronic relaxation time is also magnetic field dependent. T_{iE} are determined by the modulation of the transient zero field splitting (ZFS_T) of the electronic spin states caused by the dynamic distortions of the ligand field and, according to the **Blombergen–Morgan theory**, their magnetic field dependence is given by the following equations:

$$T_{1E}^{-1} = \frac{1}{25}\Delta^2\tau_v[4S(S+1)-3]\left(\frac{1}{1+\omega_S^2\tau_v^2} + \frac{4}{1+4\omega_S^2\tau_v^2}\right) \tag{7}$$

$$T_{2E}^{-1} = \frac{1}{50}\Delta^2\tau_v[4S(S+1)-3]\left(3+\frac{5}{1+\omega_S^2\tau_v^2} + \frac{2}{1+4\omega_S^2\tau_v^2}\right) \tag{8}$$

where Δ^2 is the square of the transient ZFS_T energy (in s^{-2}) and τ_v is the correlation time related to its modulation.

The **second-sphere term**, r_1^{SS}, describes the contribution arising from water molecules held in the proximity of the surface of the metal complex by hydrogen bonding interactions with polar groups of the ligand. By using the same approach described above for the inner sphere contribution and by assuming that the residence lifetime of the second sphere water protons is significantly shorter than their relaxation time, r_1^{SS} is given by:

$$r_1^{SS} = \frac{n_1 \cdot [C]}{55.6} \cdot \frac{1}{T_{1M}^{Hss}} \tag{9}$$

where n_1 is the number of second sphere water molecules and T_{1M}^{Hss} is the longitudinal relaxation time of the second-sphere water protons.

Analogously to the inner-sphere contribution T_{1M}^{Hss} is magnetic field dependent:

$$\frac{1}{T_{1M}^{Hss}} = \frac{2}{15}\frac{\gamma_H^2 g_e^2 \mu_B^2 S(S+1)}{r_{ss}^6}\left[\frac{3\tau_{c1}^{ss}}{1+\omega_H^2(\tau_{c1}^{ss})^2} + \frac{7\tau_{c2}^{ss}}{1+\omega_S^2(\tau_{c2}^{ss})^2}\right] \tag{10}$$

where r_{ss} is the average distance between the metal ion and the second-sphere water protons and τ_{ci}^{ss} (i =1,2) is the correlation time associated to the motion of the second-sphere water molecules.

For simplicity τ_{ci}^{ss} are given by:

$$(\tau_{ci}^{ss})^{-1} = (\tau^{ss})^{-1} + T_{iE}^{-1} \tag{11}$$

where τ^{ss} is related with the motions (reorientation or exchange) of the second sphere water protons.

The *outer sphere* term, r_1^{Hos}, describes the contribution from water molecules which diffuses around the paramagnetic complex and, according to the **model** developed by **Hwang and Freed**, may be related to the

minimum distance between the metal and the outer-sphere water protons, *a*, the relative solute–solvent diffusion coefficient, *D*, and, again, the electronic relaxation times, T_{iE} :

$$r_{1p}^{Hos} = C^{os} S(S+1)[C]\left(\frac{1}{aD}\right)[7\mathrm{J}(\omega_{\mathrm{S}}) + 3J(\omega_H)] \quad (12)$$

where C^{os} is a constant and the dependence on the electronic relaxation times is expressed in the non-Lorentzian spectral density functions $J(\omega_i)$.

Reference:

1. Relaxometry of water–metal ion interactions. In *Advances in Inorganic Chemistry*; Eldik, R. van, Bertini, I., Eds.; Academic Press, 2005 ISBN 9780080458182.
